# Supplementary material for: Comparative Genomics of Transisthmian Damselfishes (Abudefduf saxatilis and A. troschelii)
Source: Genome Biol Evol. 2026 Apr 30;18(5):evag111. doi: 10.1093/gbe/evag111 (PMC13179497; doi:10.1093/gbe/evag111)
Supplement: evag111_Supplementary_Data [file evag111_supplementary_data.docx]

**Supplemental material for:**

**Title:** Comparative Genomics of Transisthmian Damselfishes (*Abudefduf saxatilis* and *A. troschelii*)

Author List: Claire B. Tracy^1,2*^, Carlos F. Arias^2,3^, Eirlys Tysall^4,2^, Marc P. Hoeppner^5^, W. Owen McMillan^2^, Oscar Puebla^2,6,7^, Moisés A. Bernal^1, 2*^

^1^ Auburn University, Auburn, AL 36830, USA

^2^ Smithsonian Tropical Research Institute, Balboa, Ancon, Panama City, Panamá

^3^ Data Science Lab, Office of the Chief Information Officer, Smithsonian Institution, Washington, District of Columbia, USA

^4^ University of Cambridge, Cambridge, UK

^5^ Institute of Clinical Molecular Biology, Christian-Albrechts-University of Kiel, Kiel 24105, Germany

^6^ Leibniz Centre for Tropical Marine Research (ZMT), 28359 Bremen, Germany

^7^ Institute for Chemistry and Biology of the Marine Environment, *Carl von Ossietzky Universität Oldenburg,* 26111 Oldenburg, Germany

*^*^Authors for Correspondence:*

Claire B. Tracy, Department of Biological Sciences, Auburn University, Auburn, AL, USA, [cbt0022@auburn.edu](mailto:cbt0022@auburn.edu)

Moisés A. Bernal, Department of Biological Sciences, Auburn University, Auburn, AL, USA, [mab0205@auburn.edu](mailto:mab0205@auburn.edu)

Running Title: Genomics of transisthmian fishes

Keywords: *Allopatric speciation*, *geminate species, gene family expansion, genome* *evolution, marine fishes, Isthmus of Panama.*

**Supplemental Figures**

**
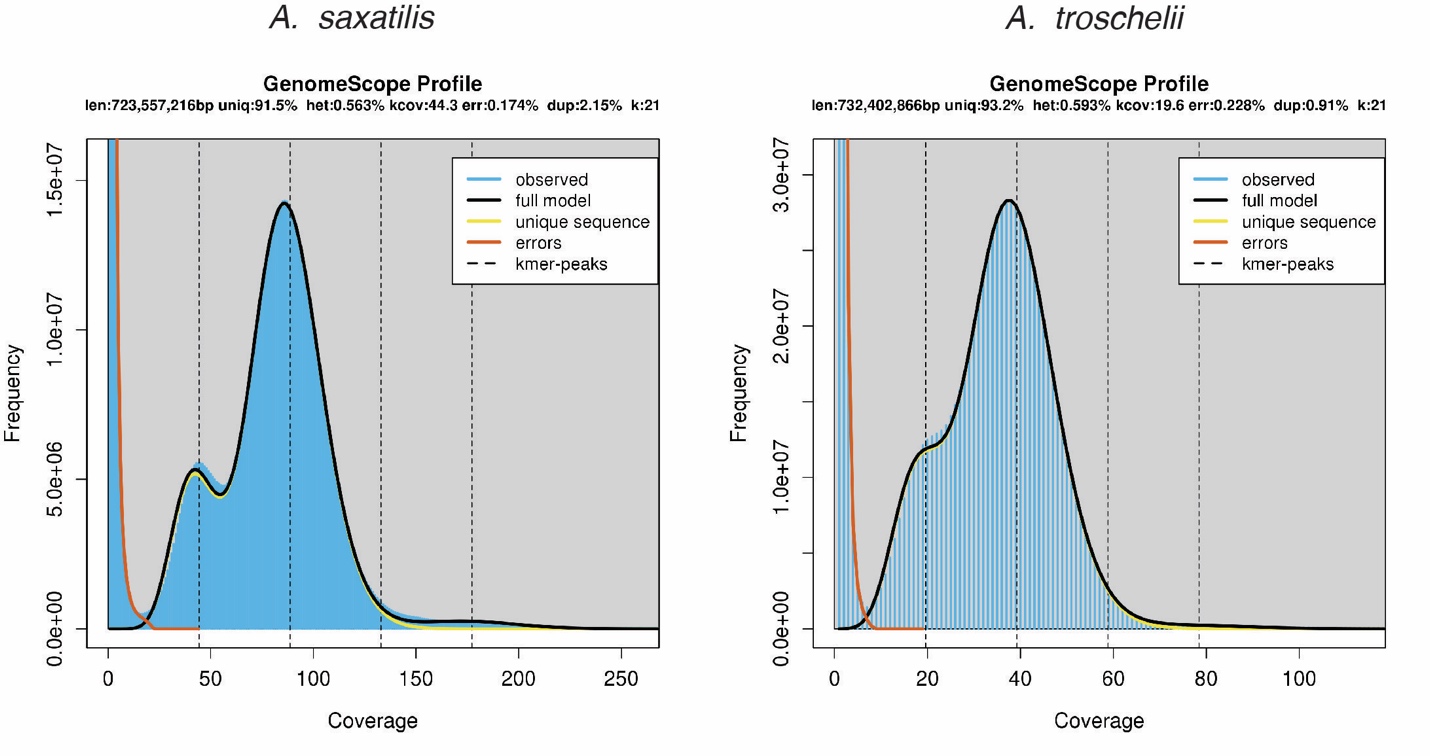
**

**Figure S1.** Frequency and coverage of the genome of A. saxatilis (left) and A. troschelii (right) from GenomeScope of the k-mer count and distribution determined using Jellyfish. The differences in coverage between the two species are from the sequencing efforts and are not associated with biological differences (see methods for more details).

**
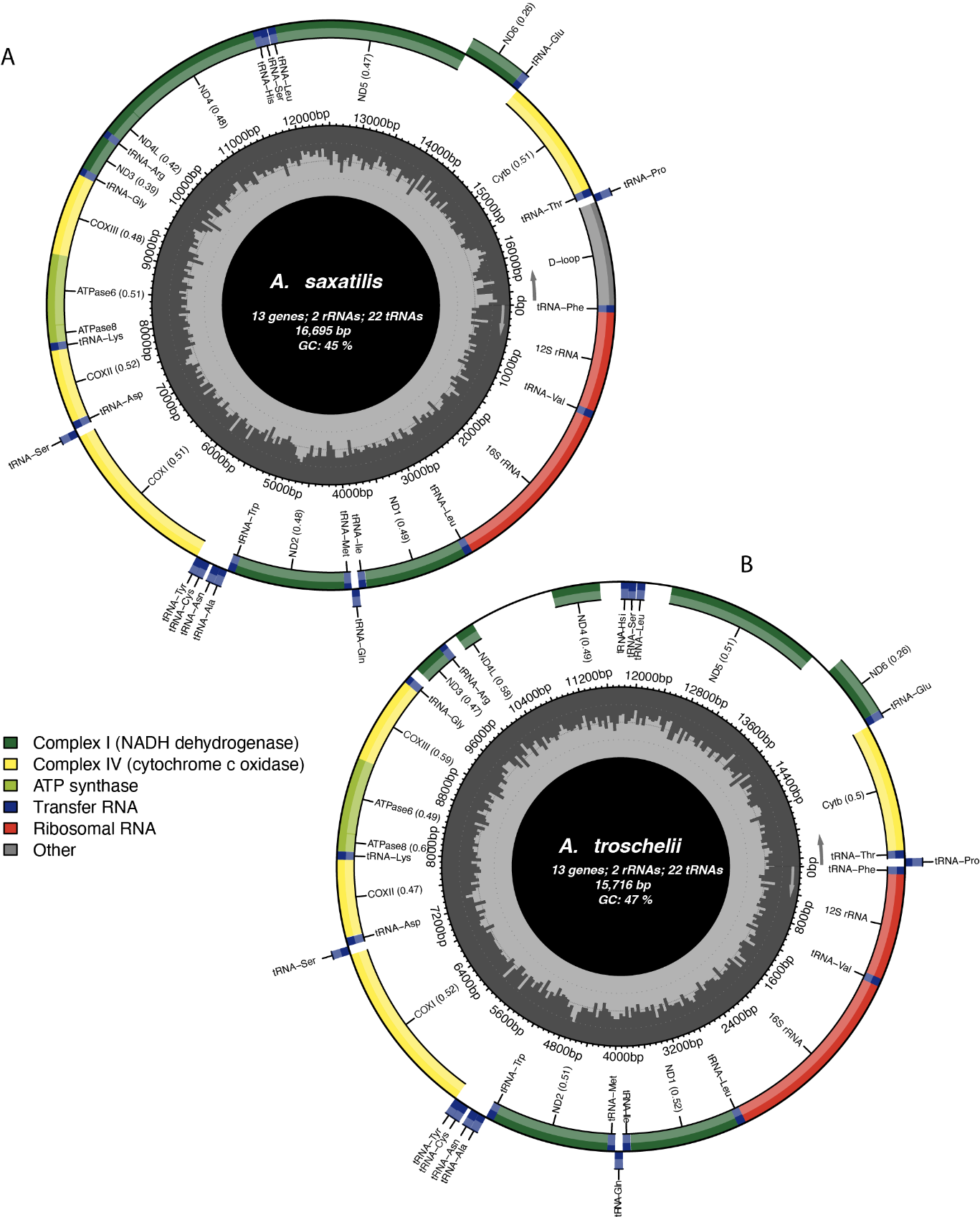
**

**Figure S2.** Annotation of the partial mitochondrial genomes of *Abudefduf saxatilis* (A) and *A. troschelii* (B), showing the coding sequences, the tRNA's and rRNA's. The images were generated using the program Chloroplot (Zheng, et al. 2020), through the MitoFish v2025.06 platform (Sato, et al. 2018).


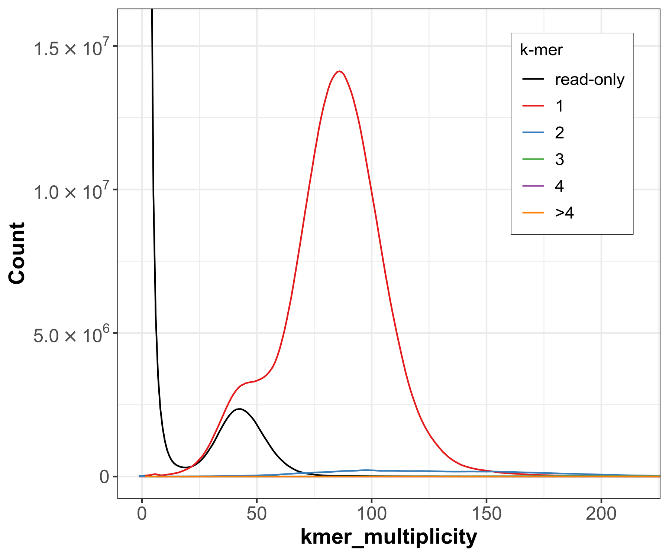

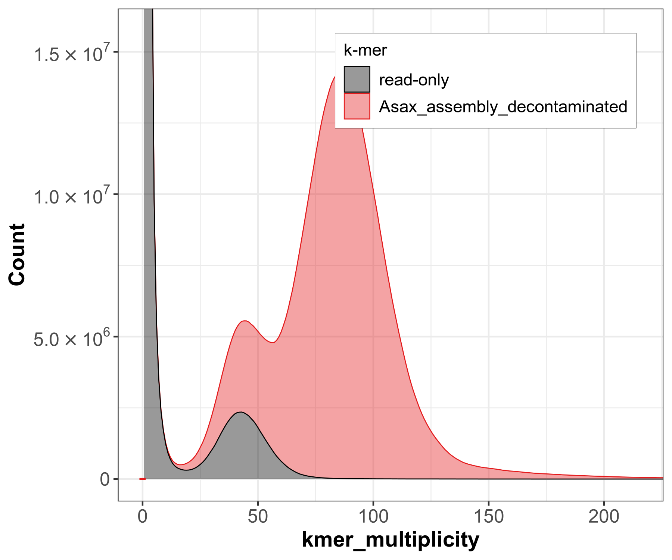


**Figure S3.** Merqury results for *A. saxatilis* showing how often k-mers from the reads are found in the assembly (left), and the distribution of k-mers from reads and assembly with their multiplicity in the reads (right).


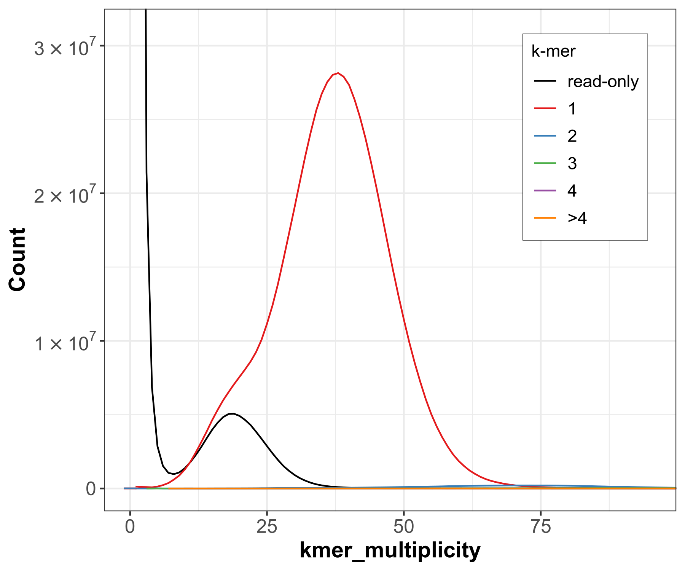

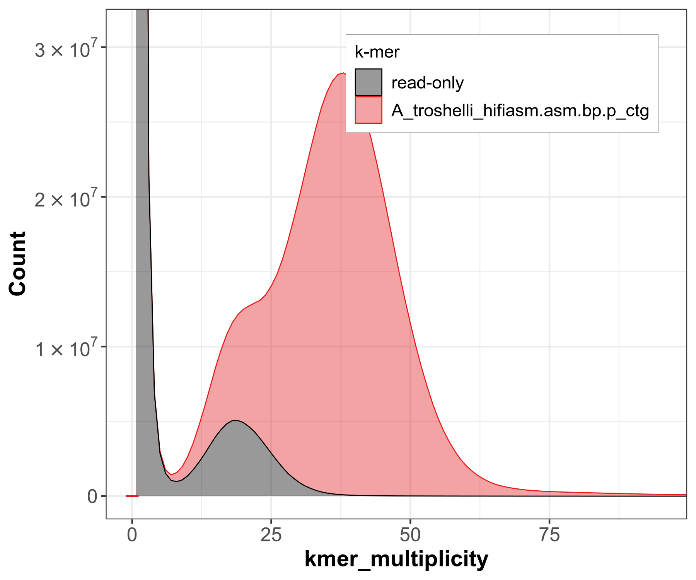


**Figure S4.** Merqury results for *A. troschelii* showing how often k-mers from the reads are found in the assembly (left), and the distribution of k-mers from reads and assembly with their multiplicity in the reads (right).


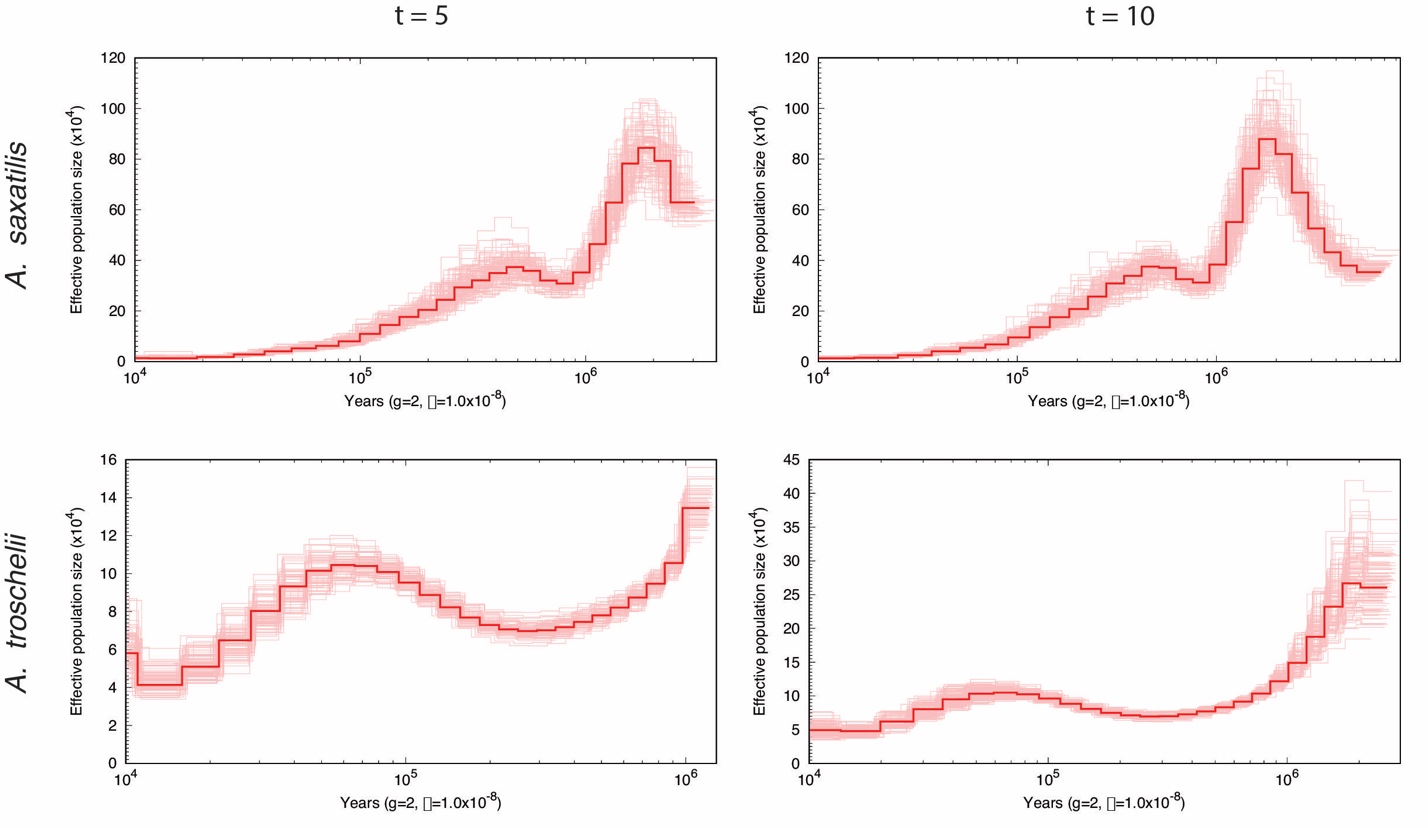


**Figure S5.** Demographic history results from PSMC for A. saxatilis (top) and A. troschelii (bottom). The analyses were made with t = 5 (left) and t = 10 (right), with 100 bootstrap replicates in each analysis. Dark red is the result from a consensus estimate, and all the light pink lines are the results from each bootstrap run. All graphs were created with an estimated generation time of 2 years. Analyses were run several times with different parameters; however the pattern of the curve did not change for either species.


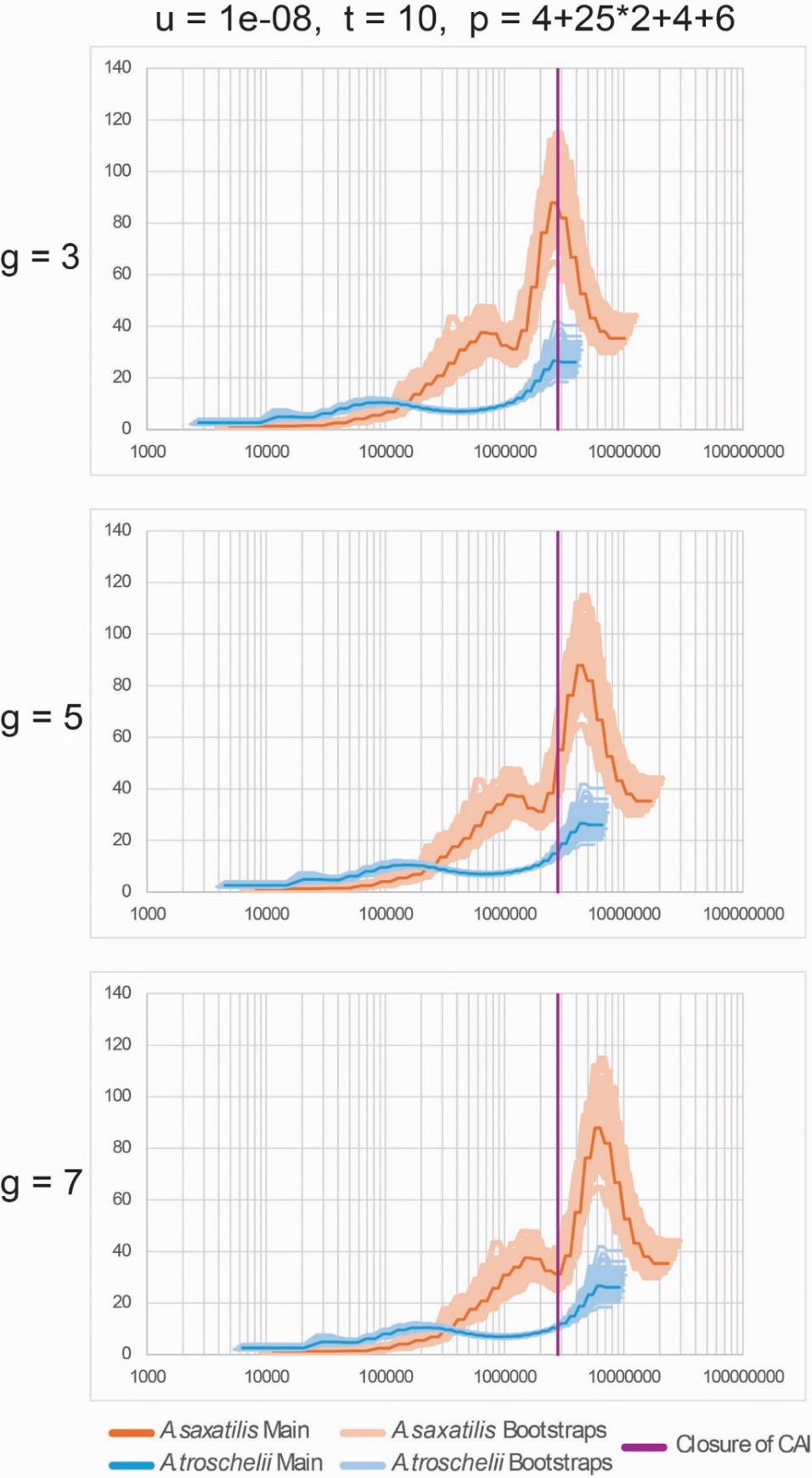


**Figure S6.** Estimates of effective population size with PSMC, changing the generation time (*g*) while keeping all other parameters constant.

**B.**

**A.**


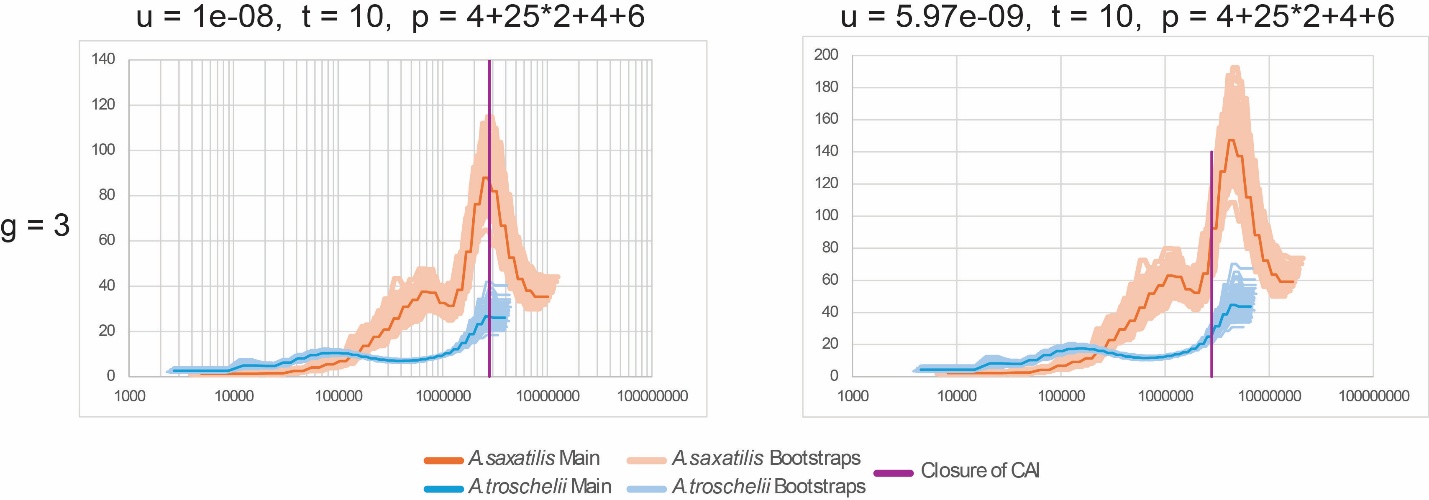


**Figure S7.** Estimates of effective population size with PSMC, changing the mutation rate while keeping all other parameters constant. Mutation rate on panel A is within the range published for *Amphiprion ocellaris* in Bergeron et al., 2023, while the mutation rate in panel B is the average for all fishes from Bergeron et al., 2023.


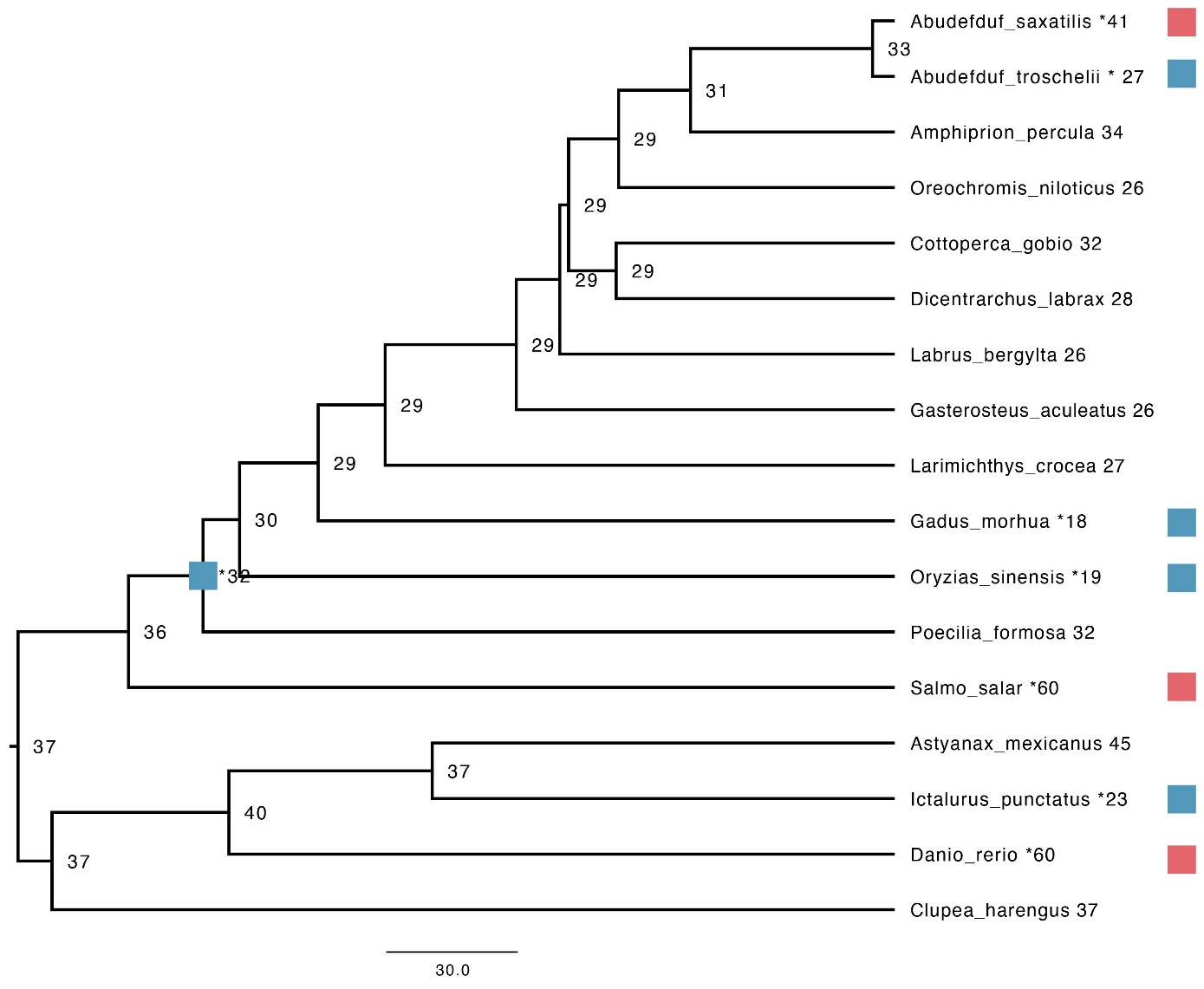


**Figure S8.** Estimates of gene family size for gamma crystallin genes across the phylogeny used in the CAFÉ analysis. Numbers to the right of each species name represents the estimated gene family size. Boxes shaded red indicate significant expansions, and boxes in blue indicate significant contractions.


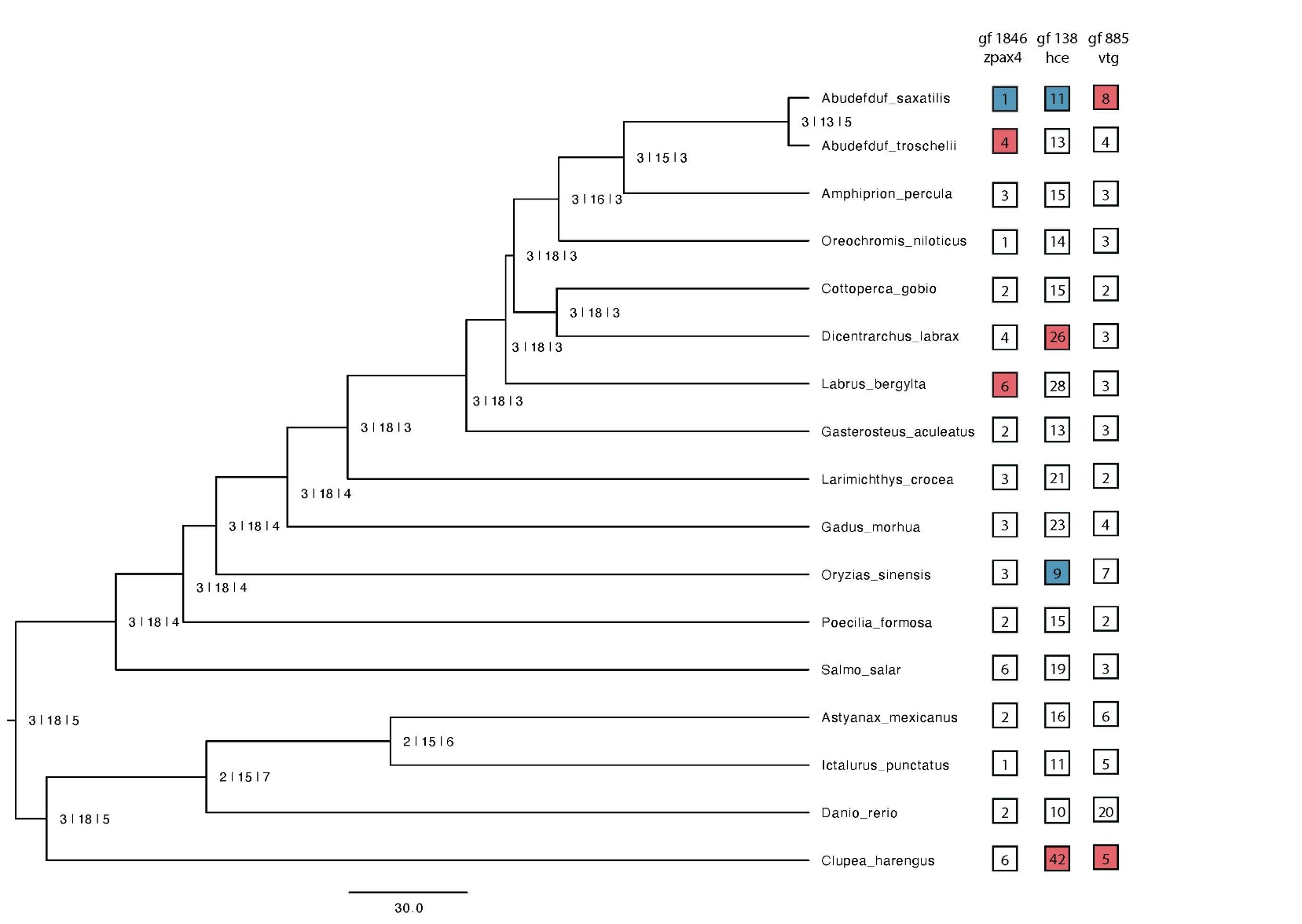


**Figure S9.** Estimates of gene family size for reproduction related genes across the entire phylogeny, using the CAFÉ analysis. The order of the gene families was: *zpax4*, *hce*, and *vtg*, and the number on the boxes represent estimated gene family size. Boxes shaded red indicate significant expansions, and boxes in blue indicate significant contractions.

**Supplemental Tables**

**Table S1.** Mapping of *A. saxatilis* contigs to Nemo genome assembly chromosomes. Contigs highlighted in red are mapped to multiple Nemo contigs, and contigs in black are mapped to only one Nemo contig.

| Nemo Contig ID | Nemo Chromosome Number | Percent Mismatch | Number mapped reads | *A.saxatilis* mapped contigs |
| --- | --- | --- | --- | --- |
| CM009708 | 1 | 2.9 | 4 | ptg000023l |
|  |  |  |  | ptg000040l |
|  |  |  |  | ptg000120l |
|  |  |  |  | ptg000138l |
| CM009709 | 2 | 3 | 3 | ptg000007l |
|  |  |  |  | ptg000034l |
|  |  |  |  | ptg000046l |
| CM009710 | 3 | 4.2 | 9 |  |
|  |  |  |  | ptg000027l |
|  |  |  |  | ptg000029l |
|  |  |  |  | ptg000062l |
|  |  |  |  | ptg000026l |
|  |  |  |  | ptg000067l |
|  |  |  |  | ptg000025l |
|  |  |  |  | ptg000034l |
|  |  |  |  | ptg000053l |
|  |  |  |  | ptg000092l |
|  |  |  |  |  |
| CM009711 | 4 | 3.2 | 4 | ptg000008l |
|  |  |  |  | ptg000015l |
|  |  |  |  | ptg000033l |
|  |  |  |  | ptg000053l |
| CM009712 | 5 | 3.1 | 2 | ptg000014l |
|  |  |  |  | ptg000045l |
| CM009713 | 6 | 4.5 | 8 |  |
|  |  |  |  | ptg000072l |
|  |  |  |  | ptg000040l |
|  |  |  |  | ptg000067l |
|  |  |  |  | ptg000026l |
|  |  |  |  | ptg000034l |
|  |  |  |  | ptg000053l |
|  |  |  |  | ptg000039l |
|  |  |  |  | ptg000077l |
| CM009714 | 7 | 2.9 | 4 | ptg000002l |
|  |  |  |  | ptg000021l |
|  |  |  |  | ptg000030l |
|  |  |  |  | ptg000077l |
| CM009715 | 8 | 3.1 | 2 | ptg000036l |
|  |  |  |  | ptg000039l |
| CM009716 | 9 | 4.7 | 15 |  |
|  |  |  |  | ptg000005l |
|  |  |  |  | ptg000019l |
|  |  |  |  | ptg000029l |
|  |  |  |  | ptg000016l |
|  |  |  |  | ptg000039l |
|  |  |  |  | ptg000053l |
|  |  |  |  | ptg000041l |
|  |  |  |  | ptg000032l |
|  |  |  |  | ptg000004l |
|  |  |  |  | ptg000058l |
|  |  |  |  | ptg000076l |
|  |  |  |  | ptg000062l |
|  |  |  |  | ptg000092l |
|  |  |  |  | ptg000038l |
|  |  |  |  | ptg000081l |
| CM009717 | 10 | 3.5 | 2 | ptg000011l |
|  |  |  |  | ptg000028l |
| CM009718 | 11 | 3 | 3 | ptg000006l |
|  |  |  |  | ptg000031l |
|  |  |  |  | ptg000038l |
| CM009719 | 12 | 2.8 | 2 | ptg000004l |
|  |  |  |  | ptg000058l |
| CM009720 | 13 | 6.5 | 14 |  |
|  |  |  |  | ptg000019l |
|  |  |  |  | ptg000062l |
|  |  |  |  | ptg000025l |
|  |  |  |  | ptg000029l |
|  |  |  |  | ptg000092l |
|  |  |  |  | ptg000026l |
|  |  |  |  | ptg000067l |
|  |  |  |  | ptg000054l |
|  |  |  |  | ptg000032l |
|  |  |  |  | ptg000077l |
|  |  |  |  | ptg000034l |
|  |  |  |  | ptg000018l |
|  |  |  |  | ptg000072l |
|  |  |  |  | ptg000003l |
| CM009721 | 14 | 3.3 | 3 | ptg000001l |
|  |  |  |  | ptg000013l |
|  |  |  |  | ptg000035l |
| CM009722 | 15 | 4.1 | 16 |  |
|  |  |  |  | ptg000018l |
|  |  |  |  | ptg000043l |
|  |  |  |  | ptg000024l |
|  |  |  |  | ptg000038l |
|  |  |  |  | ptg000092l |
|  |  |  |  | ptg000062l |
|  |  |  |  | ptg000016l |
|  |  |  |  | ptg000076l |
|  |  |  |  | ptg000053l |
|  |  |  |  | ptg000026l |
|  |  |  |  | ptg000058l |
|  |  |  |  | ptg000004l |
|  |  |  |  | ptg000032l |
|  |  |  |  | ptg000041l |
|  |  |  |  | ptg000039l |
|  |  |  |  | ptg000005l |
| CM009723 | 16 | 67.4 | 31 |  |
|  |  |  |  | ptg000009l |
|  |  |  |  | ptg000044l |
|  |  |  |  | ptg000038l |
|  |  |  |  | ptg000129l |
|  |  |  |  | ptg000173l |
|  |  |  |  | ptg000226l |
|  |  |  |  | ptg000271l |
|  |  |  |  | ptg000151l |
|  |  |  |  | ptg000166l |
|  |  |  |  | ptg000177l |
|  |  |  |  | ptg000192l |
|  |  |  |  | ptg000197l |
|  |  |  |  | ptg000227l |
|  |  |  |  | ptg000235l |
|  |  |  |  | ptg000259c |
|  |  |  |  | ptg000154c |
|  |  |  |  | ptg000248l |
|  |  |  |  | ptg000141l |
|  |  |  |  | ptg000268l |
|  |  |  |  | ptg000027l |
|  |  |  |  | ptg000182l |
|  |  |  |  | ptg000053l |
|  |  |  |  | ptg000039l |
|  |  |  |  | ptg000025l |
|  |  |  |  | ptg000092l |
|  |  |  |  | ptg000029l |
|  |  |  |  | ptg000062l |
|  |  |  |  | ptg000019l |
|  |  |  |  | ptg000026l |
|  |  |  |  | ptg000067l |
|  |  |  |  | ptg000088l |
| CM009724 | 17 | 3.6 | 1 | ptg000010l |
| CM009725 | 18 | 3 | 3 | ptg000016l |
|  |  |  |  | ptg000020l |
|  |  |  |  | ptg000034l |
| CM009726 | 19 | 2.9 | 2 | ptg000017l |
|  |  |  |  | ptg000107l |
| CM009727 | 20 | 3 | 3 | ptg000012l |
|  |  |  |  | ptg000035l |
|  |  |  |  | ptg000080l |
| CM009728 | 21 | 2.9 | 2 | ptg000008l |
|  |  |  |  | ptg000050l |
| CM009729 | 22 | 3 | 3 | ptg000003l |
|  |  |  |  | ptg000041l |
|  |  |  |  | ptg000065l |
| CM009730 | 23 | 4.1 | 13 |  |
|  |  |  |  | ptg000019l |
|  |  |  |  | ptg000016l |
|  |  |  |  | ptg000005l |
|  |  |  |  | ptg000039l |
|  |  |  |  | ptg000053l |
|  |  |  |  | ptg000041l |
|  |  |  |  | ptg000032l |
|  |  |  |  | ptg000004l |
|  |  |  |  | ptg000058l |
|  |  |  |  | ptg000076l |
|  |  |  |  | ptg000062l |
|  |  |  |  | ptg000092l |
|  |  |  |  | ptg000038l |
| CM009731 | 24 | 28 | 23 |  |
|  |  |  |  | ptg000062l |
|  |  |  |  | ptg000026l |
|  |  |  |  | ptg000025l |
|  |  |  |  | ptg000077l |
|  |  |  |  | ptg000003l |
|  |  |  |  | ptg000034l |
|  |  |  |  | ptg000019l |
|  |  |  |  | ptg000072l |
|  |  |  |  | ptg000018l |
|  |  |  |  | ptg000040l |
|  |  |  |  | ptg000067l |
|  |  |  |  | ptg000016l |
|  |  |  |  | ptg000005l |
|  |  |  |  | ptg000039l |
|  |  |  |  | ptg000053l |
|  |  |  |  | ptg000041l |
|  |  |  |  | ptg000032l |
|  |  |  |  | ptg000092l |
|  |  |  |  | ptg000004l |
|  |  |  |  | ptg000058l |
|  |  |  |  | ptg000076l |
|  |  |  |  | ptg000038l |
|  |  |  |  | ptg000024l |

**Table S2**. Descriptions for genes expanded/contracted in either *A. saxatilis*, *A. troschelii*, or at the node representing the MRCA of the two species. Summary descriptions are blank where we did not have an ID or description for the genes within that gene family.

| Family ID | Sig at MRCA | Sig in Atro | Sig in Asax | Summary Description |
| --- | --- | --- | --- | --- |
| 19 | Not Sig | Sig | Sig | protein tyrosine phosphatase |
| 20 | Sig | Sig | Not Sig | olfactoy receptor |
| 25 | Not Sig | Not Sig | Sig | serine protease |
| 26 | Not Sig | Not Sig | Sig | gap junction protein, connexin |
| 28 | Sig | Not Sig | Sig | novel immune-type recepor |
| 42 | Sig | Not Sig | Sig | cytochrome P450, family 2 |
| 44 | Not Sig | Not Sig | Sig | G-protein coupled receptor P2Y receptor family membrane, etc. |
| 45 | Sig | Not Sig | Sig | odorant receptor, family E |
| 46 | Sig | Not Sig | Sig | major histocompatibility complex class I |
| 51 | Not Sig | Sig | Sig | crystallin, gamma M |
| 52 | Not Sig | Sig | Not Sig | igfn1.1-1.4, titin |
| 54 | Not Sig | Not Sig | Sig | si:ch, si:dkey |
| 66 | Sig | Not Sig | Not Sig | si:ch, si:dkey |
| 80 | Sig | Sig | Not Sig | homeodomain interacting protein kinase (hipk), dual-specificity tyrosine-phosphorylation regulated kinase |
| 83 | Not Sig | Sig | Not Sig | udp-gal, udp-glcNAc |
| 88 | Not Sig | Not Sig | Sig | solute carrier family 22 members |
| 89 | Not Sig | Sig | Sig | immunoglobulin light constant/variable |
| 91 | Not Sig | Sig | Sig | si:ch, si:dkey |
| 95 | Sig | Not Sig | Not Sig | calcium channel, votage-dependent |
| 96 | Sig | Not Sig | Sig | cerebellin |
| 98 | Not Sig | Not Sig | Sig | udp glucuronosyltransferase families |
| 99 | Sig | Not Sig | Not Sig | si:ch, si:dkey |
| 102 | Not Sig | Not Sig | Sig | odorant receptor, families (multiple) |
| 103 | Sig | Not Sig | Not Sig | NONE |
| 107 | Not Sig | Sig | Sig | dynein, axonemal, heavy chains |
| 109 | Not Sig | Not Sig | Sig | serpin peptidase inhibitor, clades (multiple) |
| 115 | Not Sig | Sig | Sig | guanylate cyclases, natriuretic peptide receptors |
| 117 | Not Sig | Sig | Sig | histone cluster 1 H2A family members (multiple) |
| 119 | Sig | Not Sig | Sig | immunoglobulin heavy variables |
| 123 | Not Sig | Sig | Not Sig | otogelin-like; mucin |
| 125 | Not Sig | Sig | Sig | potassium voltage-gated channel, shaker-related subfamily |
| 127 | Not Sig | Sig | Not Sig | Histone H3.2, H3 histone family |
| 133 | Not Sig | Sig | Sig | actinin; spectrin |
| 134 | Sig | Not Sig | Not Sig | NONE |
| 136 | Not Sig | Sig | Sig | histones |
| 138 | Not Sig | Not Sig | Sig | hatching enzyme 1, tandem duplicates; six-cysteine containing astacin protease; high choriolytic enzyme |
| 140 | Not Sig | Sig | Not Sig | NONE |
| 142 | Not Sig | Sig | Not Sig | piggyBac transposable element derived |
| 161 | Not Sig | Sig | Not Sig | odorant receptor, family F |
| 162 | Sig | Not Sig | Not Sig | histone 1, H4, like |
| 166 | Sig | Not Sig | Not Sig | sulfotransferase families |
| 177 | Not Sig | Not Sig | Sig | si:ch, si:dkey |
| 181 | Not Sig | Not Sig | Sig | si:ch, si:dkey |
| 201 | Not Sig | Sig | Not Sig | chemokine (C-C motif) ligands |
| 219 | Not Sig | Sig | Not Sig | hairy and enhancer of split-related; hairy-related 4, tandem duplicates |
| 226 | Not Sig | Not Sig | Sig | si:ch, si:dkey |
| 236 | Not Sig | Not Sig | Not Sig | tumor necrosis factor receptor superfamily |
| 246 | Not Sig | Not Sig | Not Sig | si:ch; fucoletin-like |
| 250 | Sig | Not Sig | Not Sig | coagulation factor, collagen, integrin |
| 256 | Not Sig | Not Sig | Sig | polymeric immunoglobulin receptor like |
| 265 | Not Sig | Not Sig | Not Sig | si:ch |
| 273 | Sig | Not Sig | Not Sig | arachidonate 5-lipoxygenase |
| 283 | Not Sig | Sig | Sig | ASAX: SMOC1 (PFAMs=Kazal_2,SPARC_Ca_bdg,Thyroglob_assoc,Thyroglobulin_1; em_desc=SPARC related modular calcium binding 1); CCDC177 (Coiled-coil domain containing 177); PLEKHD1 (em_desc=Pleckstrin homology domain containing, family D (with coiled-coil domains) member 1); **ATRO: TRBV25-1 (em_desc=Immunoglobulin C-Type) x ~5; Immunoglobulin V-Set** |
| 284 | Sig | Not Sig | Sig |  |
| 297 | Sig | Sig | Not Sig | si:ch; si:dkey; histone H1 like |
| 301 | Sig | Not Sig | Not Sig |  |
| 302 | Not Sig | Sig | Not Sig | si:ch; icam3: intercellular adhesion molecule 3 |
| 320 | Sig | Not Sig | Sig | si:dkey; zgc |
| 333 | Not Sig | Not Sig | Sig | collagens |
| 336 | Not Sig | Sig | Not Sig | cathepsin, nothepsin, napsin, renin |
| 337 | Not Sig | Not Sig | Sig | si:ch; si:dkey; nxpe3: neurexophilin and PC-esterase domain family, member 3 |
| 344 | Not Sig | Sig | Sig | perforin |
| 347 | Not Sig | Not Sig | Not Sig | inter-alpha-trypsin inhibitor heavy chains |
| 354 | Sig | Not Sig | Sig | si:dkey |
| 358 | Not Sig | Not Sig | Sig | si:ch; si:dkey |
| 368 | Not Sig | Not Sig | Sig | serine peptidase, serine protease |
| 379 | Not Sig | Sig | Sig | si:ch; zgc |
| 380 | Sig | Sig | Not Sig | protein kinase cGMP-dependent 1-3 |
| 386 | Not Sig | Not Sig | Sig |  |
| 403 | Not Sig | Sig | Not Sig | si:ch; ecto-ADP-ribosyltransferase 4-like |
| 414 | Not Sig | Not Sig | Sig |  |
| 433 | Not Sig | Not Sig | Sig | hemoglobin, alpha embryonic/adult (hbae1, hbaa1, hbae3, hbae5, hbaa2) |
| 442 | Not Sig | Sig | Not Sig | poly [ADP-ribose] polymerase 14 |
| 461 | Not Sig | Not Sig | Sig | ST3 beta-galactoside alpha-2,3-sialyltransferase 1, 2 and 8 - involved in terminal sialyation of glycoproteins and glycolipids |
| 462 | Sig | Not Sig | Not Sig |  |
| 463 | Not Sig | Sig | Not Sig | actinoporin-like protein (apnl); delta-actitxin-Aeq1c-like |
| 501 | Sig | Sig | Not Sig |  |
| 544 | Not Sig | Sig | Not Sig | hemoglobin, beta embryonic 1-3 and adult 1-2 |
| 545 | Not Sig | Sig | Sig |  |
| 551 | Sig | Not Sig | Not Sig |  |
| 552 | Not Sig | Sig | Sig |  |
| 561 | Sig | Sig | Not Sig |  |
| 562 | Not Sig | Sig | Not Sig | major histocompatibility complex class II integral membrane alpha chain gne (mhc2a) |
| 566 | Sig | Not Sig | Sig |  |
| 580 | Sig | Not Sig | Not Sig | si:ch |
| 618 | Not Sig | Sig | Not Sig |  |
| 622 | Sig | Sig | Not Sig |  |
| 624 | Not Sig | Sig | Sig | SAMD9 - may play role in regulating cell proliferation and apoptosis; may play a role in the inflammatory response to tissue injury |
| 644 | Not Sig | Not Sig | Not Sig | pycard - mediates assembly or large signaling complexes in the inflammatory and apoptotic signaling pathways |
| 744 | Sig | Not Sig | Not Sig |  |
| 761 | Not Sig | Not Sig | Sig | heat shock protein family A member 12A-12B |
| 789 | Not Sig | Not Sig | Not Sig |  |
| 790 | Sig | Sig | Not Sig |  |
| 800 | Not Sig | Not Sig | Sig | asteroid homolog 1a, 1b - predicted to enable nuclease activity. Predicted to be involved in nucleic acid phosphodiester bond hydrolysis |
| 835 | Sig | Not Sig | Not Sig |  |
| 848 | Sig | Not Sig | Sig | T cell receptor gamma variable 1-5 |
| 865 | Not Sig | Not Sig | Sig | lectin, galactoside-binding, soluble 1, 2b, 2a (lgals2b) - may act as an autocrine negative growth factor that regulates cell proliferation |
| 885 | Not Sig | Not Sig | Sig | vitellogenin 1-7, vtg1 - transport protein and lipid from liver through blood to oocytes where it becomes part of the yolk |
| 902 | Not Sig | Not Sig | Sig | zgc |
| 920 | Not Sig | Sig | Sig | si:ch; g2e3 (ubiquitin protein ligase) |
| 937 | Sig | Not Sig | Not Sig |  |
| 1002 | Not Sig | Not Sig | Not Sig | si:dkey; zc |
| 1004 | Not Sig | Sig | Sig | acyl-CoAthioesterase 20-22 (acot20-22) - May play a role in controlling adaptive thermogenesis (from ACOT13 gene card) |
| 1029 | Not Sig | Not Sig | Sig | si:dkey; PATJ crumbs cell polarity complex component (patj) |
| 1073 | Not Sig | Not Sig | Sig |  |
| 1088 | Not Sig | Sig | Not Sig | CXADR lg-like cell adhesion module (cxadr); V-set and immunoglobulin domain containing 8a & 8b (vsig8a) |
| 1095 | Not Sig | Not Sig | Sig | myxovirus (influenze) resistance A-E (mxg, mxa, mxb,…) |
| 1107 | Not Sig | Sig | Not Sig |  |
| 1193 | Not Sig | Sig | Not Sig | si:ch |
| 1215 | Not Sig | Not Sig | Not Sig |  |
| 1225 | Not Sig | Sig | Not Sig |  |
| 1260 | Not Sig | Not Sig | Sig | cystatin-A5-like, cystatin 14a & b tandem duplicate |
| 1269 | Not Sig | Sig | Sig |  |
| 1274 | Not Sig | Sig | Sig | si:ch |
| 1322 | Not Sig | Not Sig | Sig | si:ch; von willebrand factor A domain containing 5A (vwa5a) |
| 1344 | Not Sig | Sig | Not Sig | complement factor H and H like 1-5; (cfhl; cfh) - plays role in regulation of complement activation, restricting innate defense mechanism to microbial infections |
| 1356 | Sig | Not Sig | Not Sig |  |
| 1372 | Not Sig | Sig | Sig |  |
| 1397 | Sig | Not Sig | Sig |  |
| 1446 | Not Sig | Sig | Not Sig | isocitrate dehydrogenase (NADP(+)) 1 & 2 (idh1, idh2) - plays critical role in generation of NADPH (biosynthesis pathways); may act as a corneal epithelial crystallin and may be involved in maintaining corneal epithelial transparency (by similarity) |
| 1449 | Not Sig | Sig | Not Sig | guanylate binding protein (ID from Danio rerio) |
| 1452 | Not Sig | Not Sig | Sig | danio rerio ring finger protein 213 (rnf213a, b), |
| 1573 | Not Sig | Sig | Not Sig | transmembrane protein 176l (tmem176l.1) |
| 1576 | Not Sig | Not Sig | Sig | glutathione S-transferase rho (gstr) -- oxidative stress associated |
| 1581 | Not Sig | Sig | Not Sig |  |
| 1652 | Not Sig | Not Sig | Sig | guanylate kinase 1a & 1b (guk1a, guk1b) - involved in catalyzing transfer of phosphate from ATP to GMP (essential for recycling GMP); involved in cGMP metabolism in photoreceptors (by similarity) |
| 1704 | Sig | Sig | Sig |  |
| 1724 | Not Sig | Not Sig | Sig |  |
| 1763 | Not Sig | Sig | Sig | barrier to autointegration factor 1 (banf1) |
| 1775 | Not Sig | Not Sig | Sig | TAP bindig protein, tandem duplicate (tapbp.1) - involved in MHC class 1 assembly; death-domain associated protein (daxx) - multifunctional protein (may regulate apoptosis) |
| 1782 | Not Sig | Sig | Not Sig |  |
| 1801 | Not Sig | Sig | Not Sig |  |
| 1816 | Not Sig | Not Sig | Sig |  |
| 1840 | Not Sig | Sig | Sig | dehydrogenase/reductase (SDR family) member 11a * b (dhrs11) |
| 1846 | Not Sig | Sig | Sig | zona pellucida protin AX 4 (zpax4) - reproduction/ sperm-egg binding (moises) |
| 1894 | Sig | Not Sig | Not Sig |  |
| 1910 | Not Sig | Sig | Not Sig |  |
| 1913 | Sig | Not Sig | Not Sig |  |
| 1915 | Not Sig | Not Sig | Sig |  |
| 1938 | Not Sig | Sig | Sig |  |
| 1942 | Not Sig | Not Sig | Sig |  |
| 1957 | Not Sig | Sig | Sig | si:ch, zgc |
| 1958 | Not Sig | Sig | Sig |  |
| 1966 | Not Sig | Not Sig | Sig |  |
| 1980 | Not Sig | Sig | Not Sig | ependymin related 1 (epdr1) - cell adhesion (maybe look into for development - mab) |
| 1996 | Sig | Not Sig | Sig |  |
| 2021 | Sig | Not Sig | Sig |  |
| 2022 | Sig | Not Sig | Sig |  |
| 2039 | Not Sig | Not Sig | Sig |  |
| 2053 | Not Sig | Not Sig | Sig |  |
| 2072 | Not Sig | Not Sig | Sig | signal sequence receptor, alpha (ssr1) |
| 2087 | Not Sig | Sig | Not Sig | pitrilysin metallopeptidase 1 (pitrm1) - peptide cleavage and degradation |
| 2096 | Sig | Not Sig | Not Sig |  |
| 2098 | Sig | Not Sig | Sig |  |
| 2102 | Sig | Not Sig | Sig | immunoglobulin heavy varible 1-4 (ighv1-4) |
| 2135 | Sig | Not Sig | Not Sig | si:dkey |
| 2156 | Not Sig | Not Sig | Sig | MCL1 apoptosis regulator, BCL2 family member a & b (mcl1a, mcl1b) |
| 2242 | Not Sig | Not Sig | Not Sig | SUB1 regulator of transcription a, b (sub1a, sub1b) |
| 2319 | Not Sig | Sig | Not Sig |  |
| 2321 | Not Sig | Not Sig | Sig |  |
| 2628 | Not Sig | Sig | Sig |  |
| 2667 | Not Sig | Sig | Not Sig |  |
| 2768 | Not Sig | Sig | Not Sig |  |
| 2964 | Not Sig | Sig | Sig |  |
| 2972 | Not Sig | Sig | Not Sig |  |
| 3201 | Sig | Not Sig | Not Sig |  |
| 3221 | Not Sig | Sig | Not Sig | dynein, axonemal, light intermediate chain 1 |
| 3241 | Not Sig | Not Sig | Sig | ESF1, nucleolar pre-rRNA processing protein, homolog (esf1) |
| 3312 | Sig | Sig | Not Sig |  |
| 3334 | Sig | Not Sig | Not Sig |  |
| 3359 | Not Sig | Not Sig | Sig |  |
| 3375 | Not Sig | Sig | Not Sig |  |
| 3398 | Sig | Not Sig | Not Sig |  |
| 3401 | Not Sig | Not Sig | Sig |  |
| 3434 | Sig | Sig | Not Sig | NO GENE DESCRIPTION/NAME FROM ANY OTHER PUBLISHED GENOMES; Asax annotation: PTBP1 (polypyrimidine tract binding protein) (only one of 7 genes has annotation info); ATRO annotation: SEC62 (SEC62 homolog, preprotein translocation factor) |
| 3581 | Not Sig | Not Sig | Sig |  |
| 3589 | Not Sig | Sig | Sig |  |
| 3602 | Not Sig | Not Sig | Sig | excision repair cross-complementation group 4 - ercc4 |
| 3655 | Not Sig | Sig | Not Sig |  |
| 3677 | Not Sig | Sig | Not Sig |  |
| 3681 | Sig | Not Sig | Not Sig |  |
| 3810 | Not Sig | Sig | Sig | transmembrane protein 123 (tmem123) |
| 3839 | Sig | Not Sig | Not Sig |  |
| 3999 | Not Sig | Sig | Sig | SEC62 homolog; pre-protein translocation factor |
| 4034 | Not Sig | Sig | Not Sig |  |
| 4038 | Not Sig | Sig | Not Sig |  |
| 4040 | Not Sig | Not Sig | Sig |  |
| 4048 | Sig | Not Sig | Sig |  |
| 4070 | Not Sig | Not Sig | Not Sig |  |
| 4305 | Not Sig | Sig | Sig |  |
| 4373 | Sig | Not Sig | Sig | Family Domain Transposase from Danio Rerio gene; Asax: L1TD1: L1 transposable element RBD-like domain x2 (PFAMs: Tnp_22_dsRBD,Tnp_22_trimer,Transposase_22); DDHD1: DDHD domain containing 1b - hydrolyzes phosphatidic acid, thought to be involved in the regulation of mitochondrial dynamics; **SCARA3: Scavenger receptor class A - protection from oxidative stress;** K02A2.6-like (PFAMs: Asp_protease_2,PNMA,RVT_1,Retrotrans_gag,SCAN,rve,zf-H2C2; PFAM:PF00078,PFAM:PF00098,PFAM:PF14893,PFAM:PF17919); SecA DEAD-like domain (em_PFAMs=AHH,Helicase_C,SEC-C,SecA_DEAD,SecA_PP_bind,SecA_SW) |
| 4543 | Not Sig | Sig | Sig |  |
| 4572 | Not Sig | Sig | Sig |  |
| 4663 | Sig | Not Sig | Not Sig |  |
| 5086 | Not Sig | Sig | Not Sig |  |
| 5160 | Not Sig | Sig | Not Sig |  |
| 5744 | Sig | Sig | Not Sig |  |
| 6814 | Not Sig | Sig | Not Sig |  |
| 6975 | Not Sig | Sig | Not Sig |  |
| 7361 | Sig | Not Sig | Sig |  |
| 7863 | Not Sig | Not Sig | Sig |  |
| 8119 | Sig | Sig | Not Sig |  |
| 8468 | Not Sig | Not Sig | Sig |  |
| 9184 | Sig | Sig | Not Sig |  |
| 9360 | Not Sig | Sig | Sig |  |
| 9423 | Not Sig | Sig | Sig |  |
| 9717 | Not Sig | Sig | Sig |  |
| 9728 | Not Sig | Not Sig | Sig |  |
| 9729 | Not Sig | Sig | Not Sig |  |
| 9759 | Sig | Sig | Not Sig |  |
| 9859 | Not Sig | Not Sig | Sig |  |
| 9875 | Not Sig | Not Sig | Sig |  |
| 9964 | Not Sig | Sig | Not Sig |  |
| 10047 | Sig | Not Sig | Sig |  |
| 10090 | Not Sig | Sig | Not Sig |  |
| 10211 | Not Sig | Sig | Not Sig |  |
| 10266 | Not Sig | Sig | Sig |  |
| 10416 | Not Sig | Sig | Sig |  |

**Table S3**. Species included in the CAFE analysis to evaluate potential expansions or contractions of gene families among transisthmian *Abudefduf*.

| **Scientific Name** | **Ensembl ID** | **Common Name** | **Family** | **Number of Proteins Raw** | **# longest proteins** |
| --- | --- | --- | --- | --- | --- |
| *Astyanax mexicanus* | ENSAMXG | Mexican Tetra | Characidae | 41410 | 27984 |
| *Amphiprion percula* | ENSAPEG | Orange Clownfish | Pomacentridae | 35899 | 25045 |
| *Cottoperca gobio* | ENSCGOG | Channel Bull Blenny | Bovichtidae | 60811 | 22935 |
| *Clupea harengus* | ENSCHAG | Atlantic Herring | Clupeidae | 67663 | 24775 |
| *Dicentrarchus labrax* | ENSDLAG | European Bass | Moronidae | 69565 | 23993 |
| *Danio rerio* | ENSDARG | Zebrafish | Cyprinidae | 65905 | 33856 |
| *Gasterosteus aculeatus* | ENSGACG | Threespine Stickleback | Gasterosteidae | 29245 | 21065 |
| *Gadus morhua* | ENSGMOG | Atlantic Cod | Gadidae | 68853 | 24085 |
| *Ictalurus punctatus* | ENSIPUG | Channel Catfish | Ictaluridae | 39017 | 25196 |
| *Labrus bergylta* | ENSLBEG | Ballen Wrasse | Labridae | 40572 | 28105 |
| *Larimichthys crocea* | ENSLCRG | Yellow Croaker | Sciaenidae | 66805 | 23506 |
| *Oreochromis niloticus* | ENSONIG | Nile Tilapia | Cichlidae | 82099 | 29453 |
| *Oryzias sinensis* | ENSOSIG | Chinese Rice Fish | Adrianichthyidae | 54551 | 22209 |
| *Poecilia formosa* | ENSPFOG | Amazon Molly | Poeciliidae | 31637 | 23950 |
| *Salmo salar* | ENSSSAG | Atlantic Salmon | Salmonidae | 184209 | 51777 |
| *Abudefduf saxatilis* | NA | Sergeant Major Damselfish | Pomacentridae | NA | 25888 |
| *Abudefduf troschelii* | NA | Panamanian Sergeant Major | Pomacentridae | NA | 25261 |
